# Preparation of Anorthite Ceramic Coatings from Purified Aluminum Dross

**DOI:** 10.3390/ma19214646

**Published:** 2026-10-31

**Authors:** Irving C. Carreón-Mendoza, José M. Almanza-Robles, Alfredo Flores-Valdés, Stephanie Soria-Sánchez, Dora A. Cortés-Hernández, José C. Escobedo-Bocardo, Enrique Diaz Barriga-Castro, José A. Rodríguez-García

**Affiliations:** 1 Centro de Investigación y de Estudios Avanzados del IPN-Unidad Saltillo, Ave. Industria Metalúrgica 1062, Parque Industrial Saltillo-Ramos Arizpe, Ramos Arizpe C.P. 25900, Mexicostephanie.soria@cinvestav.mx (S.S.-S.); dora.cortes@cinvestav.edu.mx (D.A.C.-H.); jose.escobedo@cinvestav.edu.mx (J.C.E.-B.); 2 Centro de Investigación en Química Aplicada (CIQA), Blvd. Enrique Reyna Hermosillo 140, Saltillo C.P. 25294, Mexico; enrique.diazbarriga@ciqa.edu.mx; 3 Research Department, Universidad Politécnica de Victoria, Parque Científico y Tecnológico de Tamaulipas, Ave. Nuevas Tecnologías 5902, Ciudad Victoria C.P. 870138, Mexico; jrodriguezg@upv.edu.mx

**Keywords:** anorthite (CaAl_2_Si_2_O_8_), magnesium spinel (MgAl_2_O_4_), mullite (Al_6_Si_2_O_13_), gehlenite (Ca_2_Al_2_SiO_7_), feldspars, Al-alloys

## Abstract

This investigation details the utilization of aluminum dross as a primary precursor for the synthesis of slag-based anorthite (CaAl_2_Si_2_O_8_) ceramic. To mitigate the deleterious effects of salts and impurities contained in the dross on the final mechanical and chemical properties, a multi-step hydrothermal purification (washing) process was employed. This resulted in a refined Al_2_O_3_-MgO-SiO_2_ oxide system. The synthesized anorthite was applied as a protective layer on commercial mullite substrates to enhance resistance against degradation by molten aluminum–magnesium alloys. This study characterizes the chemical, mineralogical, and thermodynamic interactions at the interface, demonstrating that this coating provides a robust barrier through the formation of stable reaction products.

## 1. Introduction

Aluminum dross is one of the waste types generated by aluminum smelters, and its disposal is the primary concern for manufacturing industries. The dross that forms on the surface of molten aluminum is the main byproduct of the aluminum smelting process [1]. It is reported that an average of 15 to 25 kg of aluminum dross is produced for every 100 kg of primary aluminum [2].

Aluminum dross is divided into two categories: primary aluminum dross and secondary aluminum dross. Primary dross is a white byproduct of the melting process and it is commonly known as white aluminum dross [3]; this is generally recycled in the smelter to recover residual metallic aluminum by remelting it with salt flux to minimize the oxidation of the metallic aluminum [4,5]. Secondary aluminum dross, which is the residue remaining after primary aluminum recovery, is characterized by its black color, and is therefore called black aluminum dross [3]. Specifically, this study utilizes secondary aluminum dross (commonly known as black aluminum dross), generated after primary metal recovery, as the precursor material. Its characteristic complex chemical composition includes Al_2_O_3_, metallic Al, MgAl_2_O_4_, AlN, SiO_2_, and salt fluxes (NaCl and KCl), as detailed in Table 1.

It is produced when certain alloying elements are used with salt flux. The alloy oxide present in the dross forms a durable network that traps metallic aluminum. The flux can break this network, facilitating the coalescence of aluminum droplets that sink into the aluminum bath. The composition of dross is complex; according to published articles [5,6], the main phases present in dross are Al_2_O_3_, metallic aluminum, and MgAl_2_O_4_, along with AlN, NaCl, MgO, Si and SiO_2_.

According to the literature, aluminum dross has been utilized for preparing magnesium–aluminum titanate-based ceramics, for high-temperature applications [7].

Ceramic hollow-fiber membranes have been prepared by extrusion from alumina-spinel composite powder, synthesized from low-cost aluminum dross waste, for the pretreatment of saline water [8].

To prepare particle-stabilized foam, obtaining ceramics with high porosity and uniformly distributed spherical pores from hydrolyzed secondary aluminum dross is required [9]. During rare earth oxide doping in spinel (MgAl_2_O_4_), the results showed that secondary aluminum dross can be used to produce spinel at a low cost [10]. Also, during concrete production, the addition of MgAl_2_O_4_ obtained from aluminum dross provides superior mechanical characteristics and durability [11].

In a previous work with aluminum dross, mullite–zirconia composites have been prepared from mixtures of aluminum dross and zircon, obtaining a material suitable for ceramic manufacturing [12]. Mullite is approximately made up of 72% alumina and 28% silica [13]. The thermal stability and refractory nature of mullite are superior to those of corundum in certain high-temperature structural applications [14]. Mullite has a high melting temperature and can withstand extremely high temperatures without deforming or melting. Furthermore, it exhibits low linear thermal expansion, which helps to minimize cracking and fracture problems during heating and cooling cycles [15]; due to these properties, a wide range of industries have found applications for it, including the metallurgical, glass, cement, and ceramic industries. It is used for the manufacturing refractory bricks, furnace linings, crucibles, nozzles, burner plates, and other components exposed to high temperatures and corrosive environments [16,17]. However, mullite contains silica, which is reduced by molten aluminum, leading to the degradation of the refractory [18], A compound with properties similar to feldspars is anorthite, which possesses numerous positive properties, such as a low theoretical density (approximately 2.76 g/cm^3^), low thermal conductivity (approximately 3.67 W/mK), and a low coefficient of thermal expansion (4.8⋅10^−6^/K). These characteristics make anorthite a very promising material for thermal insulation applications [19]. Although anorthite has already been used as one of the main components of many commercialized refractory bricks, it shows relatively low purity and poor mechanical properties, which limit its applications.

According to various authors, anorthite has been used in the following applications: (i) to fabricate single-phase porous anorthite ceramics using the foam and gel casting technique with α-Al_2_O_3_, CaCO_3_, and SiO_2_, which exhibit high strength and low thermal conductivity [20], (ii) to produce fluorapatite–anorthite glasses and glass–ceramics (eutectic composition) to evaluate their potential application in biomedicine [21], and, (iii) to obtain a strontium–anorthite based glass–ceramic with thermal, mechanical, and dielectric properties for high-temperature-resistant materials [22].

Anorthite has also been successfully synthesized using various waste materials and methods, as follows: (1) from construction waste and Al_2_O_3_ powders by adding AlF_3_ and MoO_3_ as a mineralizer and crystallization catalyst to obtain anorthite–mullite–corundum porous ceramics [23]; (2) from mineral raw materials such as clay, quartz, calcite, feldspar, and industrial alumina, without using high-purity chemical precursors [24]; (3) from fly ash, by transforming the ash residue into pure kaolinitic clay followed by thermal treatment [25]; (4) from solid waste and raw materials (aluminum dross, silica fume, and limestone) to prepare a stoichiometric anorthite ceramic (CaAl_2_Si_2_O_4_) for its use as low-thermal-conductivity conductors [26]; and (5) from residual-slag- [27] and copper-slag-based anorthite in order to develop iron-slag-based anorthite for preparing ceramics to utilize recycled slags in producing commercial ceramics, such as floor tiles and tableware [28].

In this work, a method for the synthesis of anorthite (CaAl_2_Si_2_O_8_) from aluminum dross was developed. Furthermore, the capacity of these synthesized anorthite coatings to enhance the corrosion resistance of mullite substrates was evaluated.

## 2. Experimental Procedure

### 2.1. Precursor Materials

Anorthite (CaAl_2_Si_2_O_8_) was synthesized from mixtures of CaCO_3_ (Jalmek, Ciudad General Escobedo, Mexico, 99%), Al_2_O_3_ (calcined alumina, Almatis, Ludwigshafen, Germany), SiO_2_ (commercial silica), and secondary aluminum dross (black aluminum dross, supplied by Mahle company, Ramos Arizpe, Mexico), which were subsequently submitted to a multi-step hydrothermal purification process.

For the preparation of the substrates, commercial-grade mullite with an excess of 15% of silica (Mullite Virginia #325 Mesh) was used. To obtain the alloys (Al-5Mg, Al-3Mg, and Al-1Mg), pure Al and Mg were used.

### 2.2. Dross Purification

A 5 kg batch of aluminum dross was prepared, which was crushed and ground in a mill with alumina pellets for 4 h. Once ground, the dross powders were sieved to pass through an ASTM #270 mesh (45 microns). Subsequently, the dross was washed in water at 100 °C for 2 h, and then filtered and dried at 70 °C for 24 h. Once purified, the dross was placed inside a mill with acetone, and the mill was placed on rotating rollers to homogenize the powders. Finally, the purified dross was analyzed by X-ray diffraction (XRD) (Phillips X Pert 3040, Amsterdam, the Netherlands) and Inductively Coupled Plasma Spectrometry (ICP) (PerkinElmer Optima 8300 ICP-OES, Shelton, CT, USA). From an environmental and safety perspective, the hot water leaching step is critical. Beyond the hydrolysis of aluminum nitride (AlN), which releases ammonia (NH_3_), the contact between secondary aluminum dross and water can trigger the decomposition of trace reactive impurities such as aluminum sulfides, phosphides, and silicides. Consequently, hazardous gases including hydrogen sulfide (H_2_S), phosphine (PH_3_), and silane (SiH_4_) may also be generated [6]. On an industrial scale, this water purification treatment requires appropriate gas scrubbing and ventilation systems to capture these gaseous emissions safely.

### 2.3. Synthesis of Anorthite Compounds

The synthesis of the compounds was carried out using the following methodology: after purifying the dross and when the chemical analysis results were obtained, stoichiometric calculations were conducted to determine the quantities of oxides added from the purified dross (Al_2_O_3_, MgO, and SiO_2_). The powder quantities were stoichiometrically adjusted to 100%, in conjunction with the corresponding oxides (CaCO_3_, Al_2_O_3_, and SiO_2_), required to obtain the anorthite compound.

Subsequently, the prepared powders were placed into plastic jars with acetone (C_3_H_6_O) and alumina pellets, and then homogenized using rotating rollers for 2 h; after, those powders were dried at 80 °C for 24 h. Pellets measuring 1 cm in diameter were conformed at a shaping pressure of 69 MPa, and, finally, the pellets were sintered; the sintering temperature for the anorthite compound (CaAl_2_Si_2_O_8_) was 1300 °C for 4 h.

After sintering, the pellets were ground with a mortar until the powders reached a particle size that passed through an ASTM #270 mesh (45 microns). These powders were analyzed by X-ray diffraction (XRD), thereby obtaining the single-phase slag-based anorthite compound.

### 2.4. Coating Preparation

The substrate was prepared using commercial mullite with excess of silica, which was formed into pellets measuring 4 cm in diameter at 69 MPa and sintered at 1450 °C for 4 h. To obtain the coatings, suspensions of the anorthite compound were prepared with 60% solid and 40% liquid (water), using 0.1% of a dispersant (maleic acid, C_4_H_4_O_4_).

These suspensions were applied to the surface of the mullite pellets using a technique based on the work of Dr. Blade. This technique involves a device where the mullite pellet (Al_6_Si_2_O_13_), used as the substrate, is inserted; the ceramic coating paste is then placed on its surface, and a metal spatula performs a sweep across the surface to achieve coatings measuring 1 mm in thickness.

**Figure 1:**
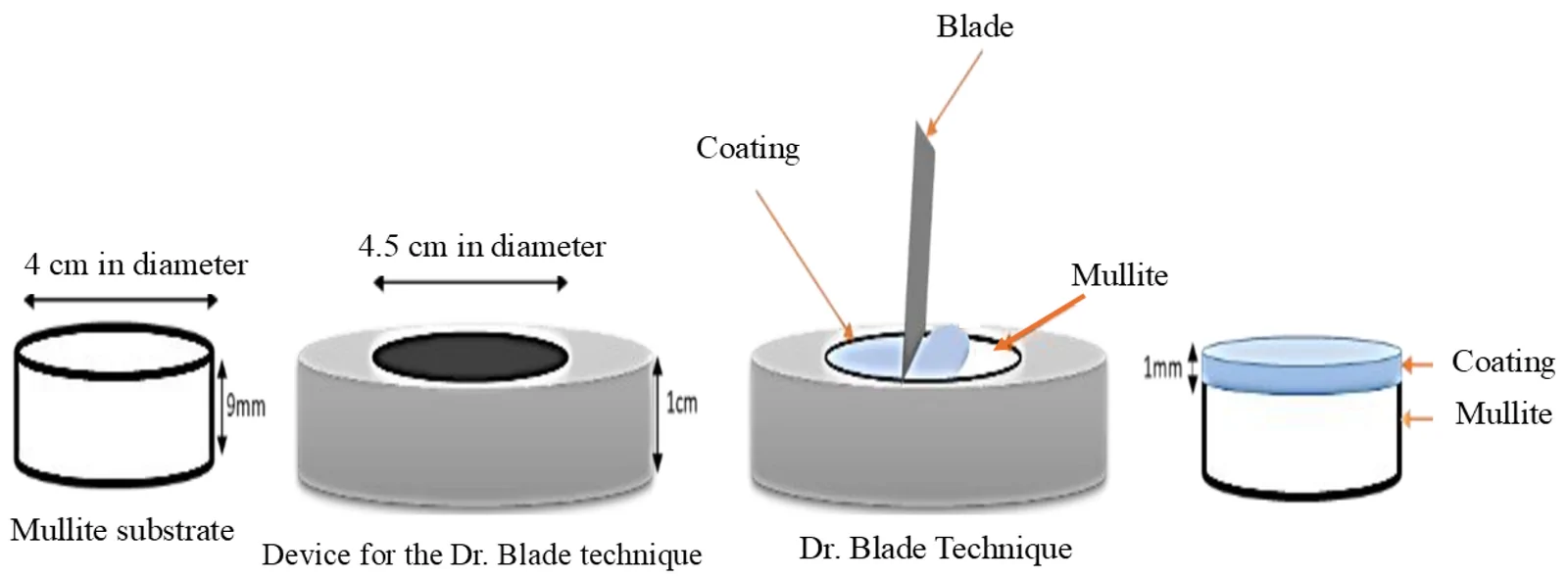
Schematic diagram of the Dr. Blade technique.

Once the coating was applied, samples were dried for 2 h at 70 °C in a drying oven and then sintered at 1300 °C for 4 h (Figure 1). Finally, the samples were cross-sectioned and analyzed using SEM (scanning electron microscopy) (Philips XL-30 ESEM, Amsterdam, the Netherlands).

### 2.5. Alloy Preparation

Al-based alloys were prepared with 1, 3, and 5 wt.% Mg; pure aluminum ingots were melted into a medium-frequency induction furnace (manufactured in Cinvestav Saltillo, Mexico). The aluminum was placed inside a graphite crucible and heated up to its melting temperature (660 °C). At that point, pure Mg was added to adjust the composition to the desired levels, and the resulting alloys were analyzed by atomic emission spectrometry by spark excitation.

### 2.6. Corrosion Testing

For corrosion testing, the coated samples (anorthite CaAl_2_Si_2_O_8_) were fixed with CaO-Al_2_O_3_ refractory cement at the bottom of the crucible. Subsequently, a charge of 400 g of the Al-5Mg alloy was placed inside the crucible, and the furnace was programmed at a temperature of 1000 °C for 100 h. Corrosion testing was only performed using only this alloy since it presents the highest corrosion rate according to preliminary trials. After the corrosion tests, samples were cross-sectioned and analyzed using SEM (scanning electron microscopy) (Philips XL-30 ESEM, Amsterdam, the Netherlands), TEM (transmission electron microscopy) (FEI Talos F200X (S)TEM, Eindhoven, the Netherlands), and XPS (X-ray photoelectron spectroscopy) (Thermo Fisher Scientific K-Alpha+ S2027, east Grinstead, UK).

### 2.7. Wettability

The wettability tests were performed using the “sessile drop” technique, where an Al alloy cylinder is placed on a substrate, heated to high temperature, and the contact angle between the liquid drop and the solid is measured. The experiment was conducted in a tubular furnace with a sight glass at one end, which allows for filming the coated pellet in contact with the alloy inside the furnace. Cylinders for the Al-Mg alloys were prepared, measuring 1 cm in diameter by 1 cm in height for each of the alloys.

**Figure 2:**
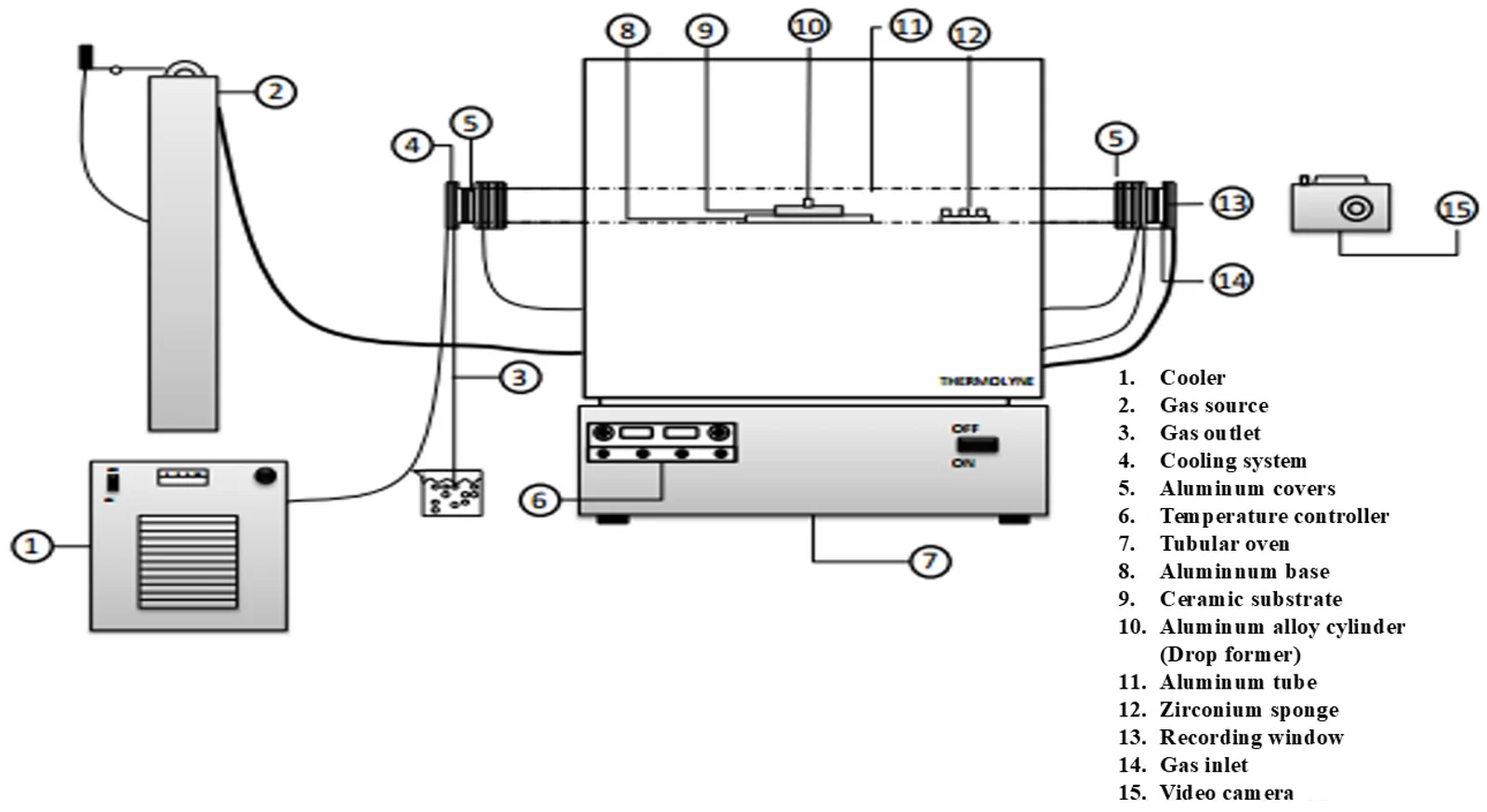
Schematic diagram of the equipment used for sessile drop tests.

Before each test, a cylinder was polished up to #2000 SiC grit paper and underwent a hydrochloric acid bath at a 1:1 ratio of water:acetone. The cylinder was then placed on the coated substrate and introduced into the tubular furnace. A metallic zirconium sponge was used inside the alumina tube to consume residual oxygen. The tube was sealed with vacuum grease and argon (Ar) was injected to prevent oxidation. A camera was placed at the end of the tube, which has a window integrated into one of the covers to record the interior. Once the desired temperature was reached, the recording was performed for 2 h, taking photographs every 5 min to measure the angle of the formed drop; contact angles were measured on both the left and right sides of the sample. Figure 2 shows the scheme of the equipment used for the test.

## 3. Results and Discussion

### 3.1. Chemical Study of the Aluminum Dross

**Figure 3:**
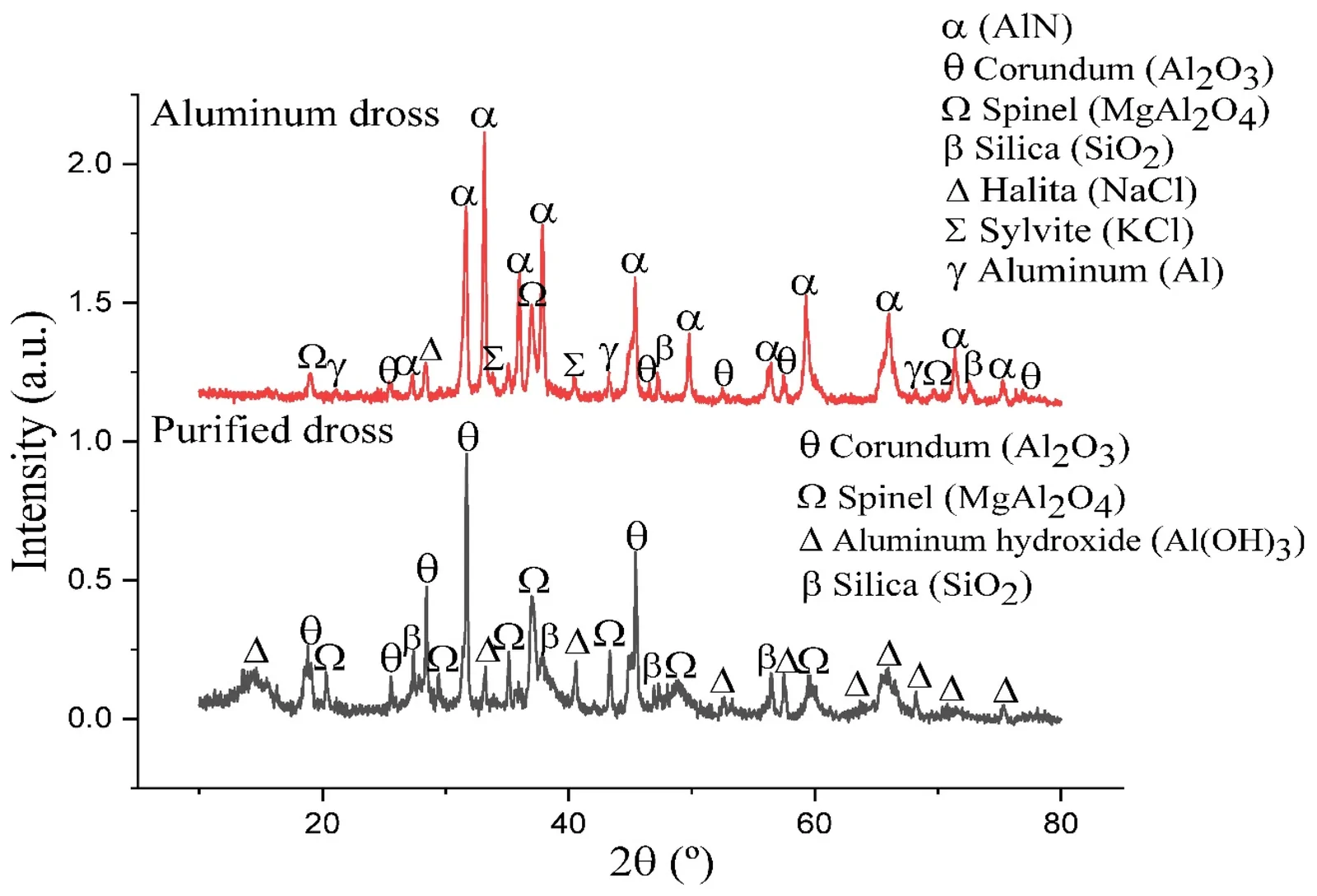
XRD patterns of unpurified and purified dross.

Figure 3 shows the diffraction patterns for the unpurified aluminum dross powders (red line), in which it is observed that the main peaks correspond to aluminum nitride (AlN) and corundum (Al_2_O_3_). Additionally, the presence of metallic aluminum (Al), spinel (MgAl_2_O_4_), and silica (SiO_2_) was detected in smaller amounts, as were impurities such as halite (NaCl) and sylvite (KCl); therefore, it was necessary to separate the main compounds of the dross through purification.

The black line shows the diffraction pattern of the purified dross, where the presence of compounds like NaCl and KCl, impurities that hinder the formation of the desired compounds, is no longer observed due to the dross washing. According to chemical analysis, impurities remain present in trace amounts, which is why they are not detected by X-ray diffraction (XRD). The oxides present (Al_2_O_3_, MgAl_2_O_4_, SiO_2_) are suitable for the formation of the intended compounds. During the dross purification process, the aluminum nitride, in contact with water and temperature, produces ammonia, decomposing the aluminum nitride; in this way, the aluminum contained in the nitride becomes part of the corundum, and aluminum hydroxide is eventually formed.

Table 1 shows the chemical analysis results, reported as oxides, of the unpurified and purified dross powders. 

This is intended to corroborate and complement the previously mentioned X-ray diffraction (XRD) results, as this analysis proves to be even more reliable. Nitrogen was not detected by the techniques used for chemical analysis (AA and ICP). Once the chemical analysis of the purified dross was obtained, the percentages of oxides (Al_2_O_3_, MgO, SiO_2_) were adjusted to 100% and, based on these amounts, stoichiometric calculations were performed to obtain anorthite (CaAl_2_Si_2_O_8_).

### 3.2. Compounds Formed from the Dross

Table 2 lists the precursors used and their stoichiometric proportion (wt.%) needed to obtain anorthite.

**Figure 4:**
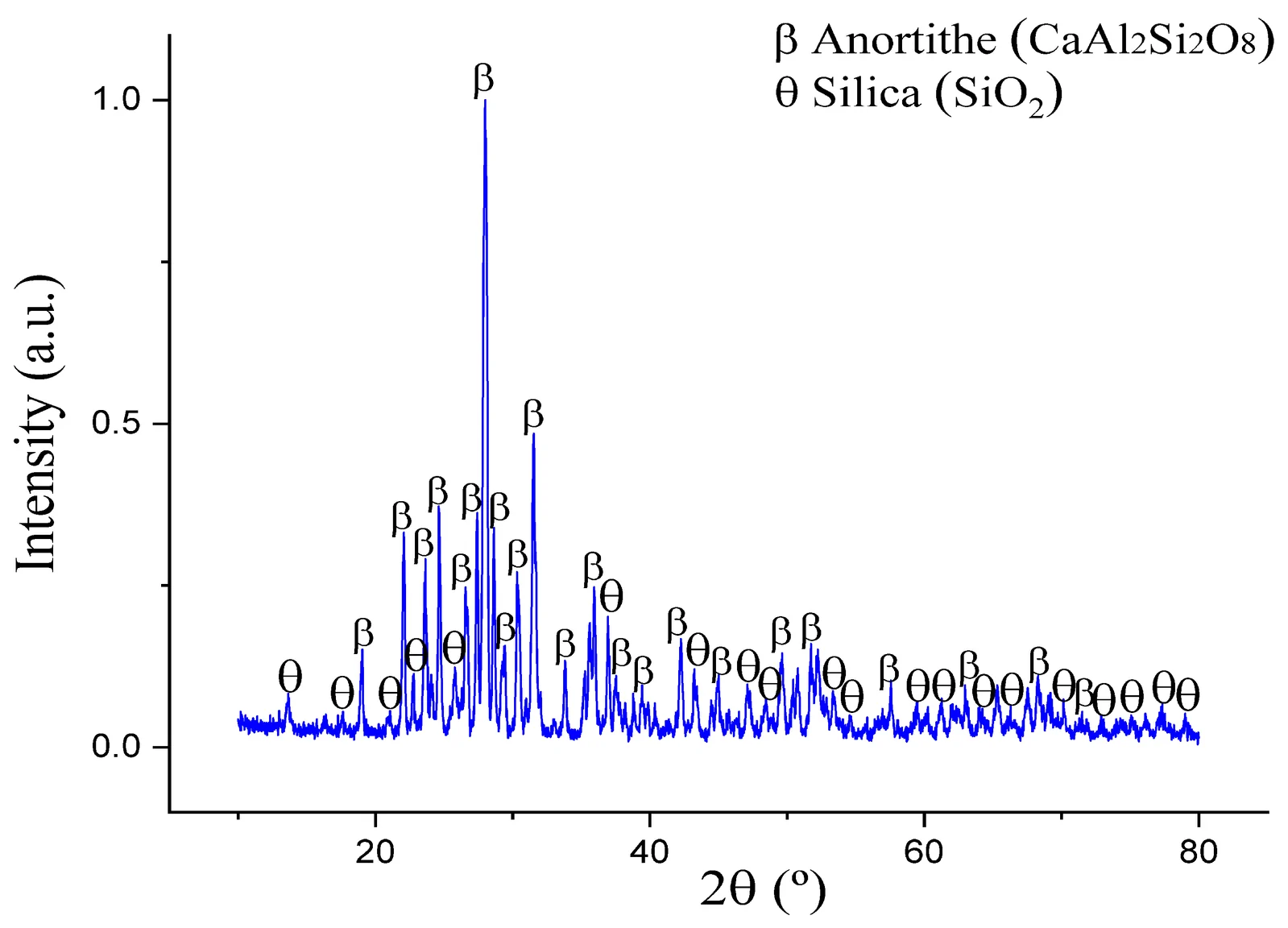
XRD pattern of the products obtained using stoichiometric amount of reagents.

Figure 4 shows the X-ray diffraction (XRD) pattern of the obtained compounds, where, apart from anorthite, a secondary phase of unreacted free silica is detected. Although the silica percentage is low, its presence may be detrimental to the compound used as a ceramic refractory, since a reduction reaction (SiO_2_ + Al = Al_2_O_3_ + Si) occurs in the presence of molten aluminum alloys, thereby degrading the refractory. Therefore, it was decided to add an excess of calcined alumina so that the silica could react and yield the desired single-phase compound.

Hamzawy [26] successfully prepared a ceramic foam based on the anorthite composition (CaAl_2_Si_2_O_8_) from solid waste containing aluminum dross, fly ash, and limestone, obtaining, at sintering temperatures between 1200 °C and 1300 °C, anorthite (~83.8% CaAl_2_Si_2_O_8_) as the main phase, along with pseudowollastonite (~9.9% Ca_3_Si_3_O_9_) and hedenbergite (~6.3% CaFeSi_2_O_6_).

In contrast, as described above, in the present work, a secondary phase of unreacted free silica (SiO_2_) was also detected. This may be due to the impurities contained in the dross. Free silica may affect the formation of anorthite; therefore, it was decided to substitute a percentage of dross with calcined alumina for the reagents so that the silica could react and yield the desired compound composition, as only the alumina contained in the dross was initially considered. The formation of anorthite as a single phase was achieved by adding calcined alumina; tests were conducted by adjusting the percentage of dross used, and compounds with 5, 10, and 15 wt.% of Al_2_O_3_ were synthetized, where the extra 15 wt.% of calcined alumina was the specific amount necessary to reach 100 wt.% of anorthite.

**Figure 5:**
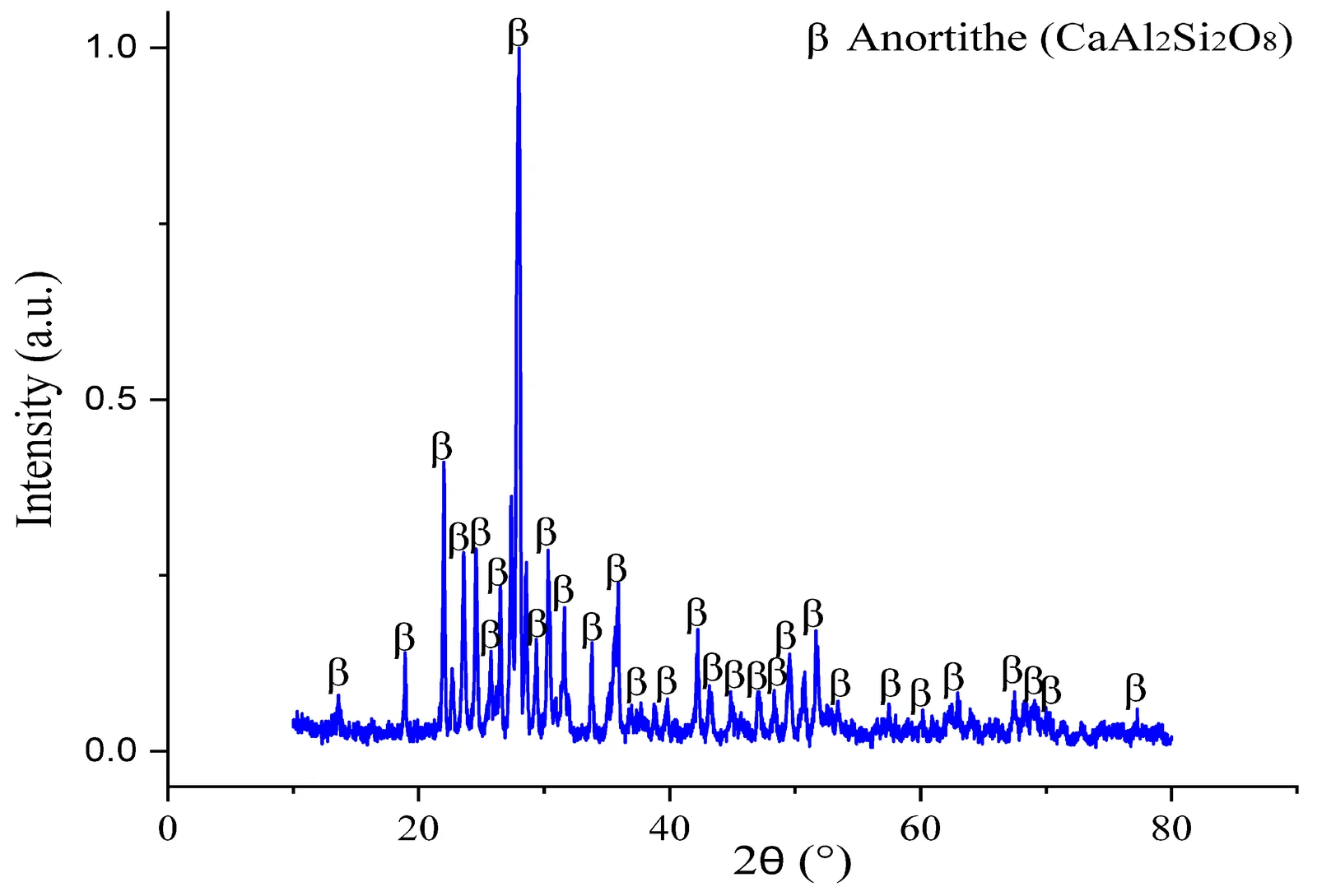
X-ray diffraction (XRD) pattern of the anorthite formed using an excess of 15 wt.% of alumina.

Figure 5 shows the diffraction pattern of the anorthite compound (CaAl_2_Si_2_O_8_) formed from the purified dross adding an excess of alumina (15 wt.%) at a temperature of 1300 °C, where only a single-phase compound can be observed. The spinel from the dross was decomposed, magnesium was replaced by calcium, and the alumina from the spinel was incorporated into the rest of the Al_2_O_3_ in the anorthite.

### 3.3. Anorthite

**Figure 6:**
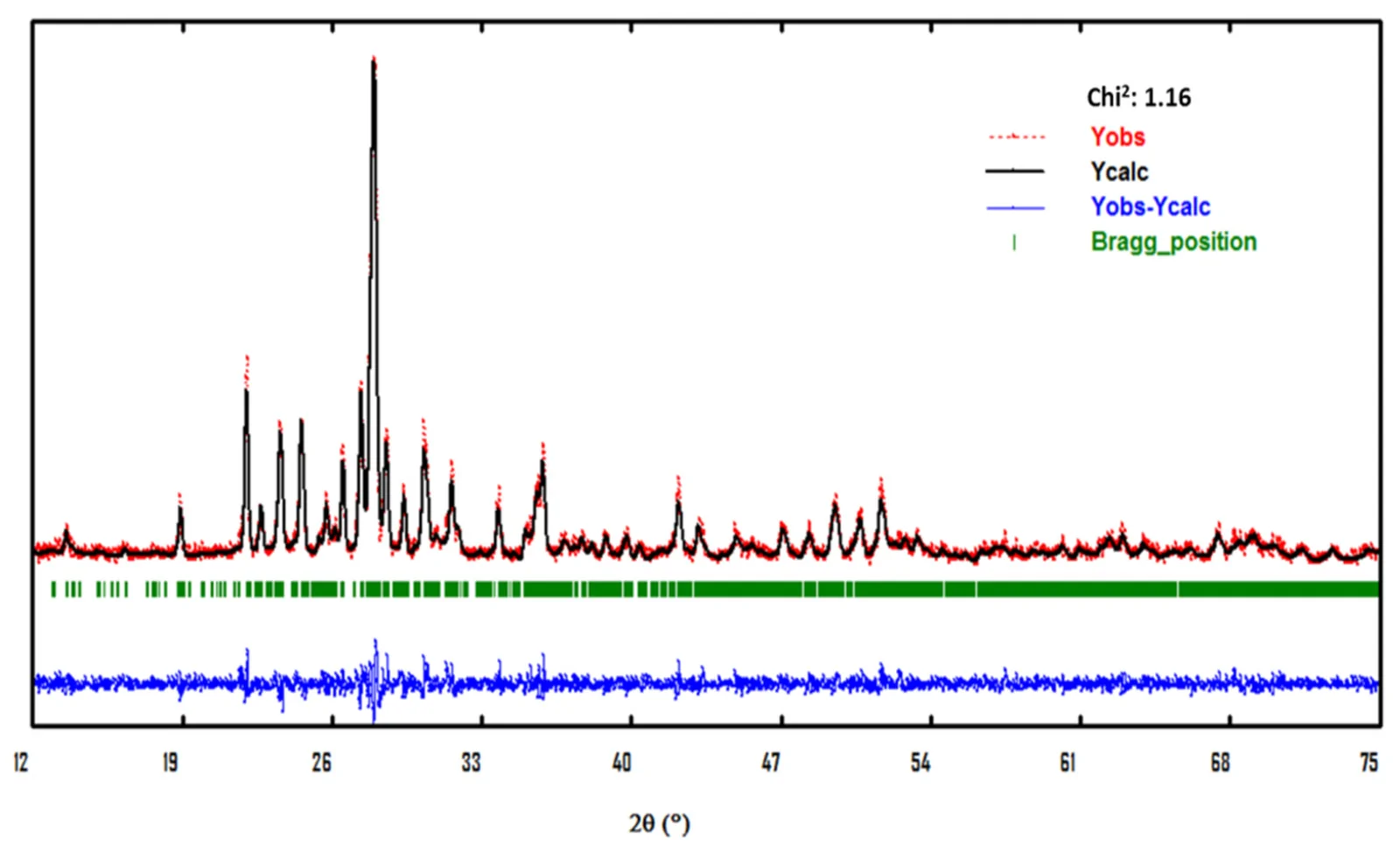
Refinement based on X-ray diffraction (XRD) data of an anorthite sample using the FullProf program.

Figure 6 shows a graph obtained after the structural refinement process, using the FullProf program based on X-ray diffraction (XRD) data (Figure 5) of an anorthite sample.

**Figure 7:**
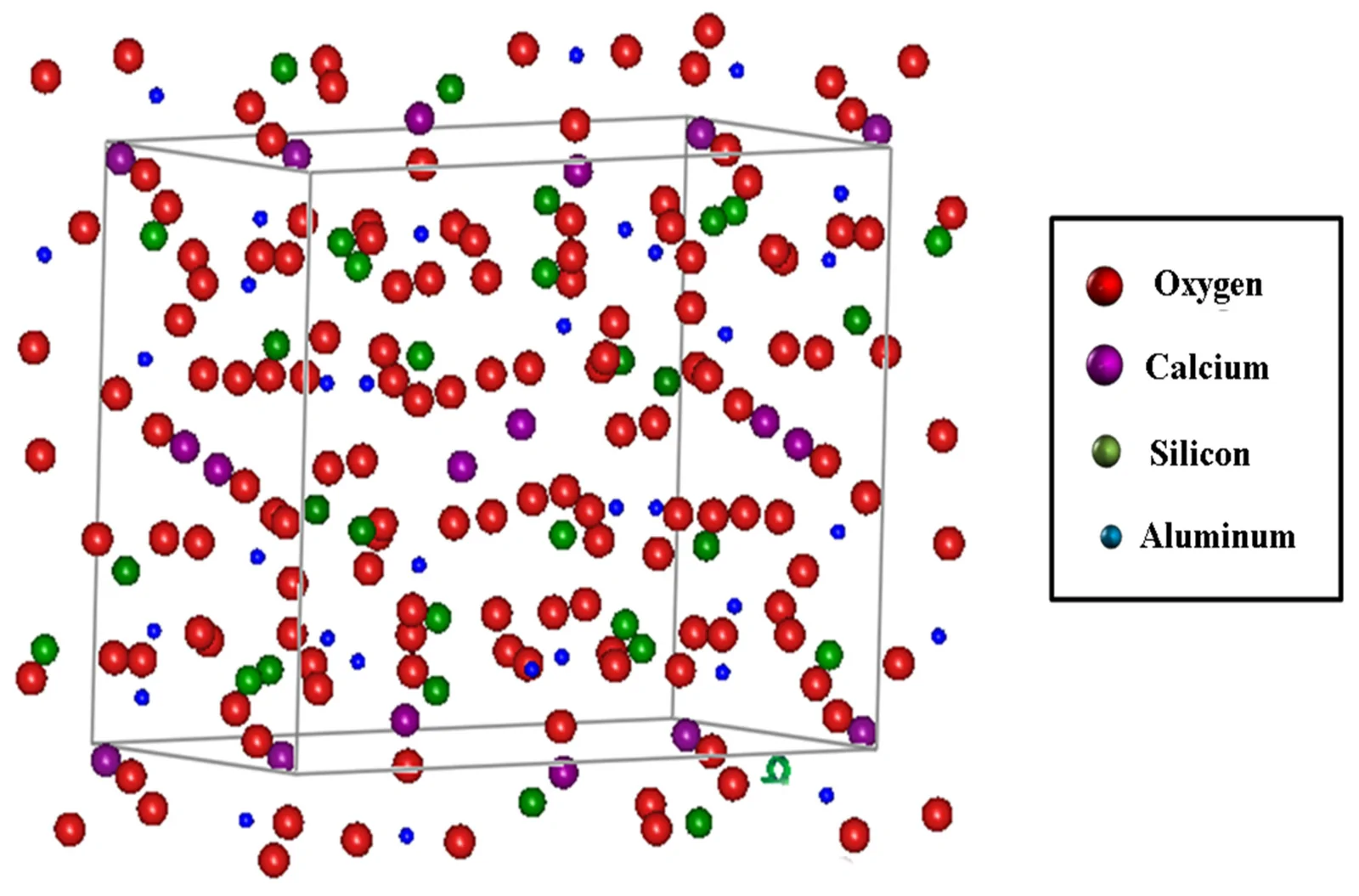
Unit cell and atomic distribution of anorthite obtained using the FullProf program.

An optimal fit can be observed between the experimental curve (red dots) and the modeled curve (black line). The appropriate fit is demonstrated by the obtained Chi^2^ value of 1.16. Based on this refinement, an analysis of the lattice parameters and the atomic distribution within the unit cell was performed; these results are presented in Figure 7.

**Figure 8:**
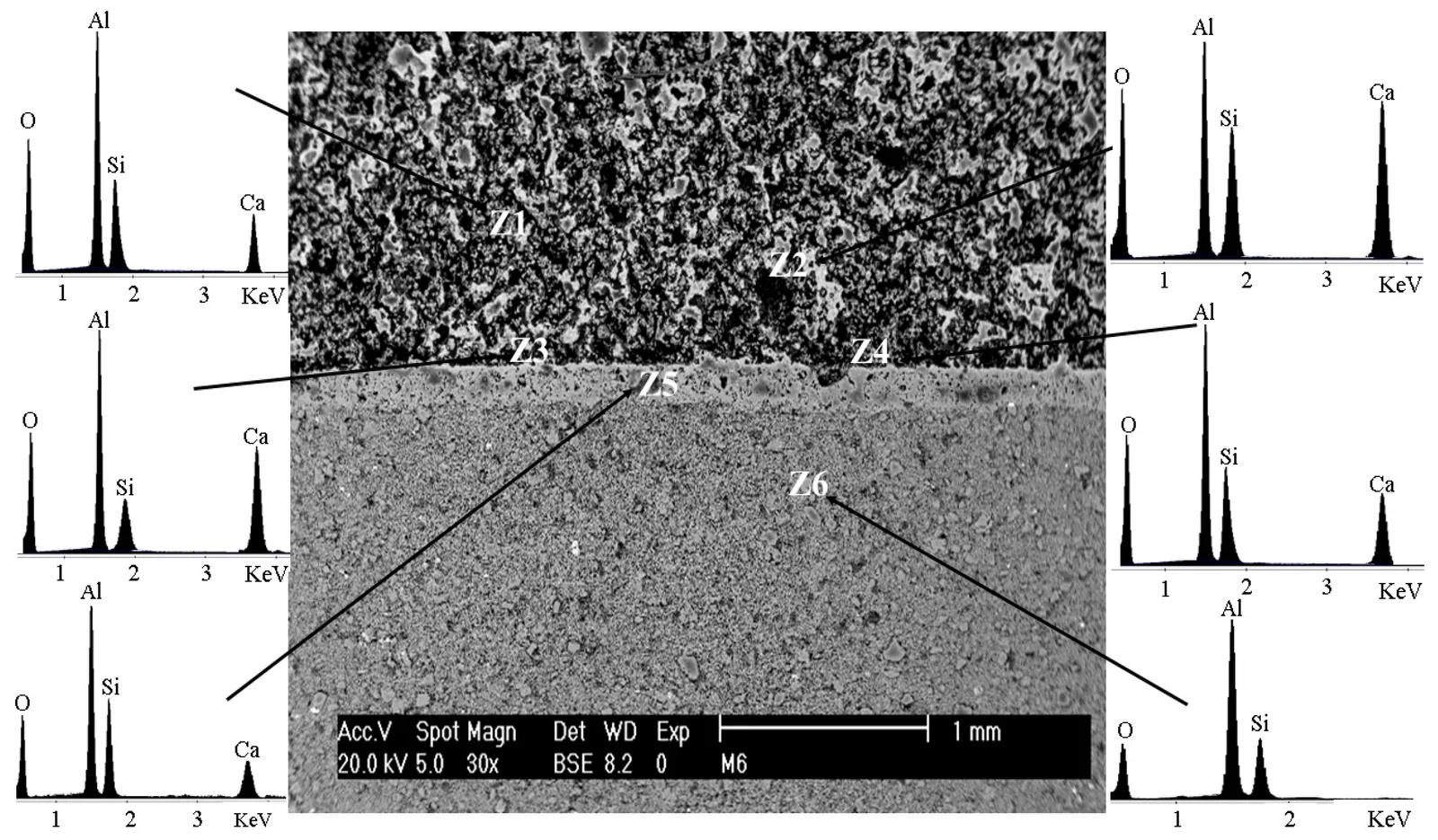
Scanning electron microscopy (SEM) image corresponding to the anorthite coating on a mullite substrate.

Figure 8 corresponds to the micrograph of the anorthite (CaAl_2_Si_2_O_8_) coating, where the main elements composing the anorthite are detected. The analysis was conducted across six zones: Zones Z1 to Z4 belong to the anorthite coating. Zone Z5 corresponds to the interface between the substrate and the coating, which is easily observable, indicating a chemical bond between them. As will be presented later (Table 3), despite thermodynamic simulations suggesting that no new reaction products may form (Al_6_Si_2_O_13_ + SiO_2_ + CaAl_2_Si_2_O_8_ = Al_6_Si_2_O_13_ + SiO_2_ + CaAl_2_Si_2_O_8_), in practice, there is compatibility between both compounds, forming a notable and properly adhered interface. Finally, zone Z6 belongs to the commercial mullite substrate. 

### 3.4. Micrographs of Coatings After Corrosion Testing

In this section, the results of the thermodynamic simulation using the FactSage^®^ 6.4 program are presented and analyzed. The proposed thermodynamic systems for the interaction between the substrate and the coatings, as well as the interaction of the compounds with the alloys, are presented and compared with the corrosion tests.

Table 3 presents the equilibrium systems for the reaction of each compound with the aluminum–magnesium alloy. Systems 1 and 2 demonstrate that mullite with excess of silica is unstable, as it degrades in the presence of metallic Al and Mg; when aluminum comes into contact with refractory elements, it produces an aluminothermic reaction, reducing the silica and producing metallic silicon and alumina (SiO_2_ +Al = Al_2_O_3_ + Si).

System 3 pertains to the reaction between commercial mullite and anorthite, where they are thermodynamically in equilibrium and form no new reaction products. Regarding Systems 4 and 5, the equilibrium reaction products show that when the anorthite coating (CaAl_2_Si_2_O_8_) comes into contact with molten aluminum and magnesium at temperatures between 800 and 1000 °C, oxide products such as silicon and alumina are obtained in some cases, indicating degradation. However, stable oxides such as spinel (MgAl_2_O_4_) and gehlenite (Ca_2_Al_2_SiO_7_) are also produced, which are resistant to degradation.

Table 4 shows the chemical composition of the obtained Al-Mg alloys.

**Figure 9:**
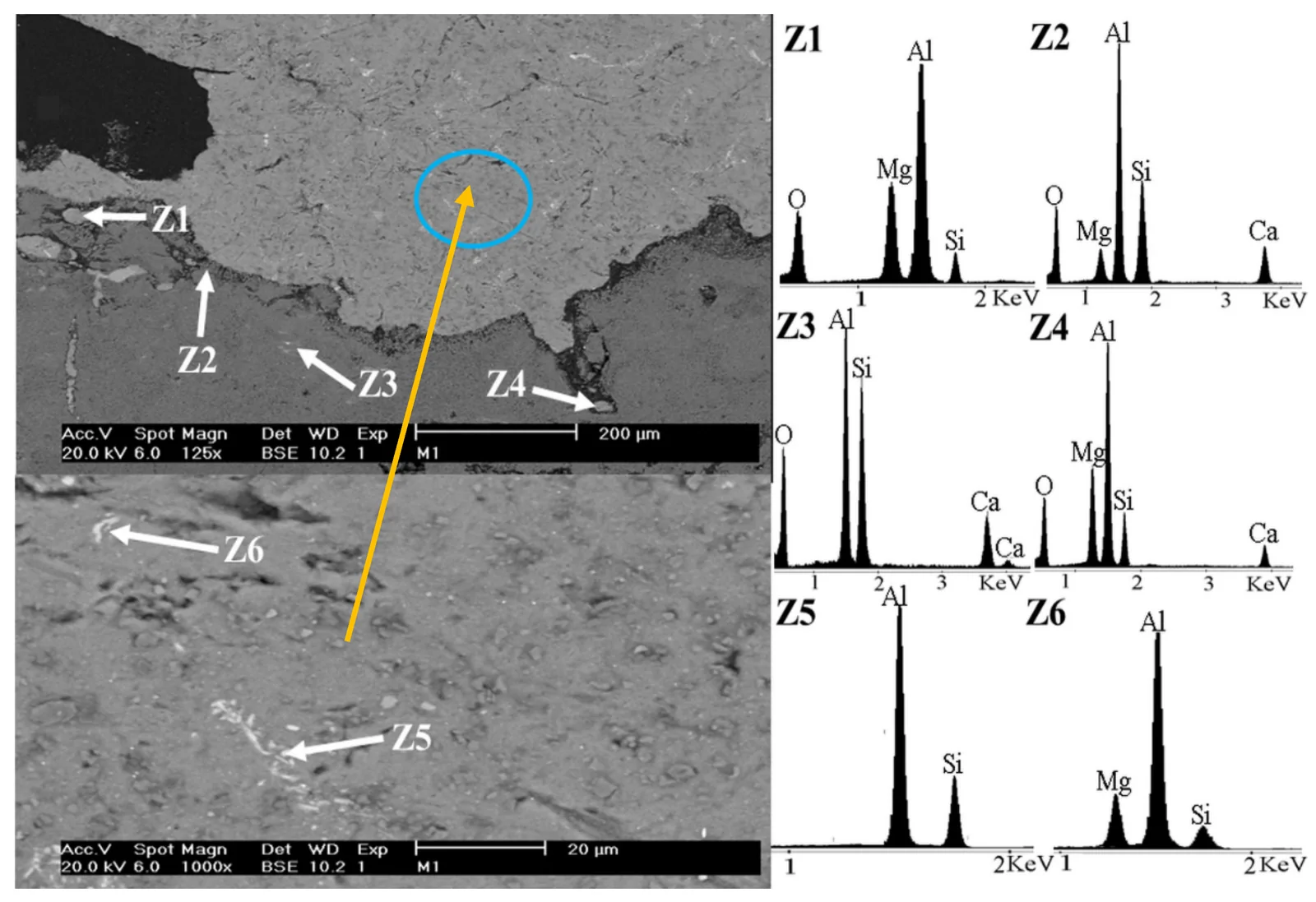
Scanning electron microscopy (SEM) images of the Al-5Mg alloy–anorthite coating interface after corrosion testing. The SEM image in the bottom is an amplification of the circled area.

Figure 9 shows to scanning electron microscopy (SEM) images of the anorthite coating (CaAl_2_Si_2_O_8_), in contact with the Al-5Mg alloy, after corrosion testing.

Zone Z1 shows the interface between the metallic aluminum alloy and the surface of the anorthite coating, where silicoaluminates and magnesium from the alloy are observed. This may indicate magnesium spinel formation.

Zones Z2, Z3 and Z4 correspond to the anorthite coating; Z2 and Z4 show the presence of magnesium from the alloy.

Zones Z5 and Z6 correspond to the Al-Mg alloy, where the presence of silicon is observed, indicating that a reduction reaction occurred.

According to the results obtained in this work, the proposed chemical reactions that may be occurring are the following:
2CaAl_2_Si_2_O_8_ + 4Al = 3Si + $\frac{9}{3}$Al_2_O_3_ + Ca_2_Al_2_SiO_7_∆G°_1000°C_ = −84.9637 kcal (1)3CaAl_2_Si_2_O_8_ + 4Al = 3Si + $\frac{12}{3}$Al_2_O_3_ + Ca_3_Al_2_Si_3_O_12_∆G°_700°C_ = −91.6976 kcal (2)2CaAl_2_Si_2_O_8_ + $\frac{10}{4}$Al + $\frac{9}{4}$Mg = $\frac{9}{4}$MgAl_2_O_4_ + Ca_2_Al_2_SiO_7_ + 3Si∆G°_1000°C_ = −131.3923 kcal (3)5CaAl_2_Si_2_O_8_ + $\frac{18}{4}$Al + $\frac{21}{4}$Mg = $\frac{21}{4}$MgAl_2_O_4_ + Ca_2_Al_2_SiO_7_ + Ca_3_Al_2_Si_3_O_12_ + 6Si∆G°_800°C_ = −283.675 kcal (4)5CaAl_2_Si_2_O_8_ + 5Al + $\frac{18}{4}$Mg = $\frac{18}{4}$MgAl_2_O_4_ + Ca_2_Al_2_SiO_7_ + Ca_3_Al_2_Si_3_O_12_ + Al_2_O_3_ + 6Si ∆G°_700°C_ = −282.205 kcal (5)

The proposed reactions exhibit negative Gibbs free energy values, which indicate that these reactions occur spontaneously. Gehlenite (Ca_2_Al_2_SiO_7_) can be formed by the reduction of CaAl_2_Si_2_O_8_ by the molten alloy. The combination of these phases may change the substrate properties, affecting corrosion rate, i.e., gehlenite shows a thermal expansion coefficient of 7 × 10^−6^/°C. The difference between the coefficients of the coating (anorthite, 4.4 × 10^−6^/°C) and the reaction products can create microcracks, allowing aluminum to infiltrate increasing corrosion. Conversely, when anorthite comes into contact with the molten alloy, the formation of magnesium spinel (MgAl_2_O_4_) is observed. This is an equilibrium reaction product that is beneficial for the coating, as it provides degradation resistance properties.

**Figure 10:**
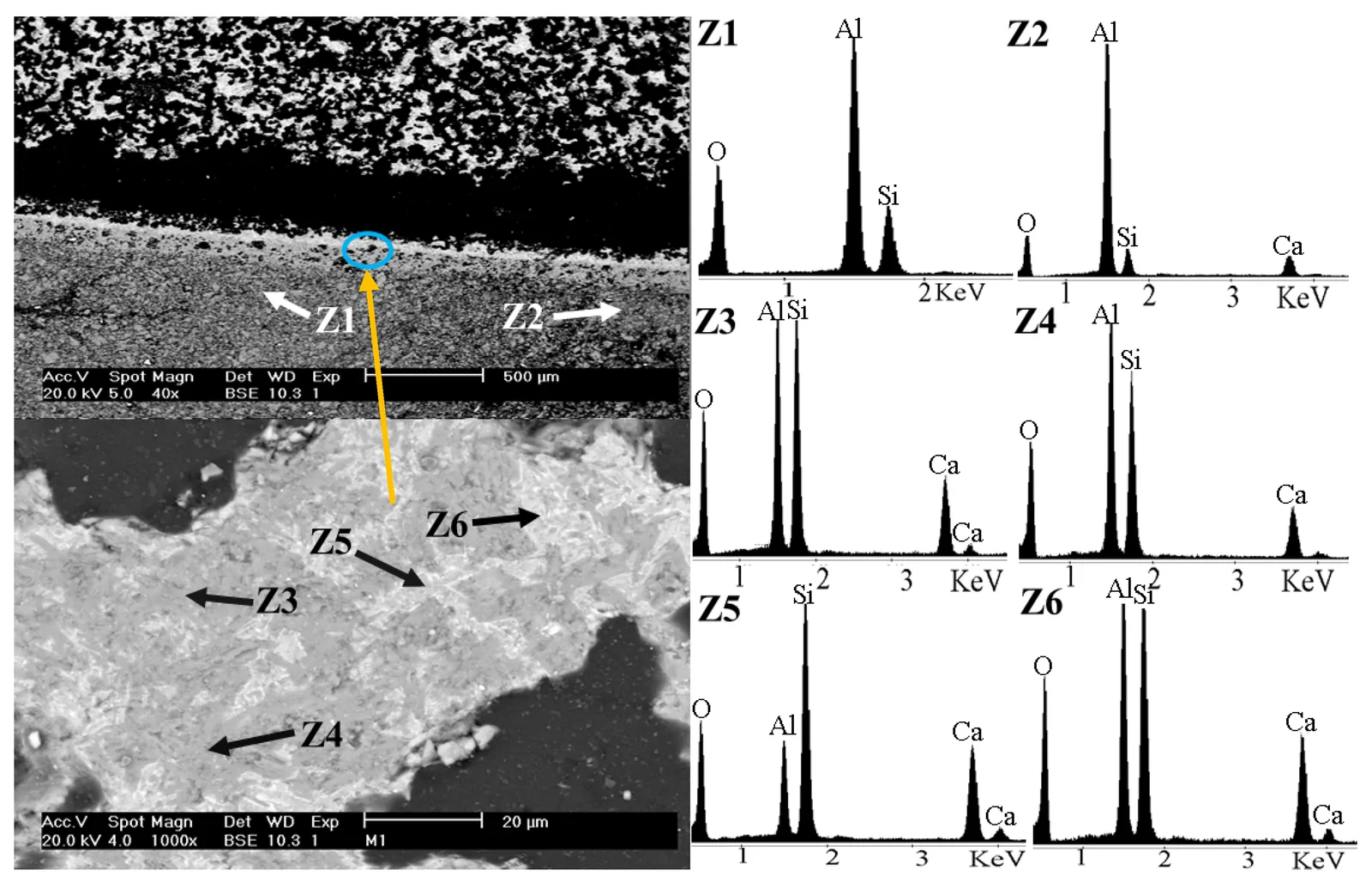
Scanning electron microscopy (SEM) images of the interface between the anorthite coating and the mullite substrate after corrosion testing. The SEM image in the bottom is an amplification of the circled area.

Figure 10 corresponds to scanning electron microscopy (SEM) images of the anorthite coating (CaAl_2_Si_2_O_8_) in contact with the mullite substrate at the interface zone after corrosion testing. Zones Z1 and Z2 correspond to the mullite substrate. The presence of calcium was found in Z2, which diffused through the substrate–coating interface, indicating that a chemical bond occurred. No presence of Al or Mg was observed, meaning that the coating protected the substrate adequately. Zones Z3 and Z4 correspond to the gray part of the coating matrix, where the oxides may correspond to the anorthite compound. Zones Z5 and Z6 correspond to a white area of the coating matrix that contains a higher amount of calcium and silicon than other zones; therefore, the formation of gehlenite (Ca_2_Al_2_SiO_7_) may have occurred in these areas.

### 3.5. Selected Area Electron Diffraction (SAED)

**Figure 11:**
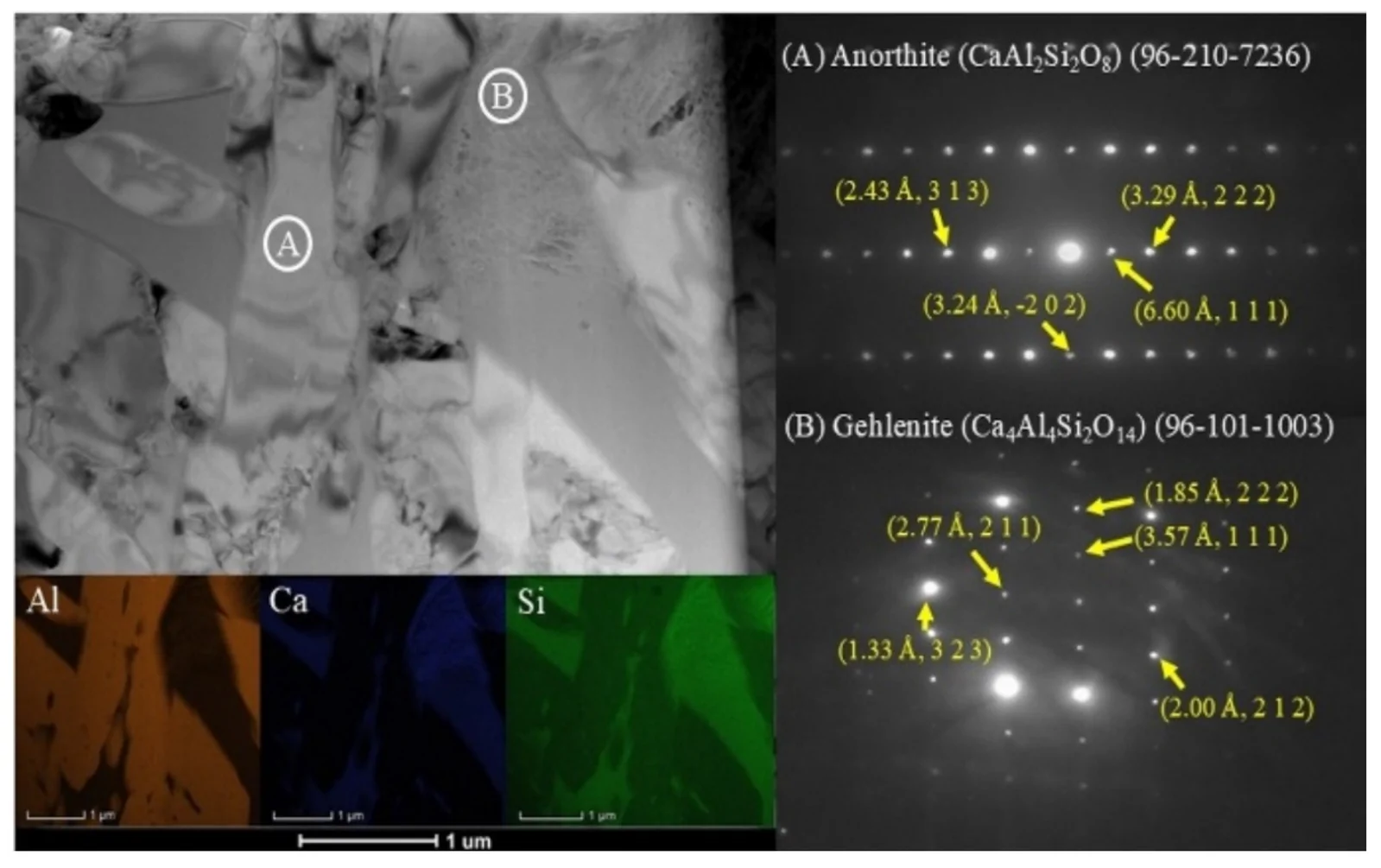
Transmission electron microscopy (TEM) analysis of the anorthite surface.

Figure 11 shows the transmission electron microscopy (TEM) results of the anorthite coating. In the A zone, the measured interplanar distances correspond to the single-phase anorthite compound. In contrast, the B zone corresponds to the gehlenite compound, which was formed by the reduction reaction of anorthite by molten aluminum on the anorthite coating during the corrosion test.

While authors such as Adabifiroozjaei et al. [29] reported the formation of gehlenite (Ca_2_Al_2_SiO_7_), calcium monoaluminate (CA), calcium dialuminate (CA_2_), alumina (Al_2_O_3_), and magnesium spinel (MgAl_2_O_4_) in an anorthite system in contact with a molten Al alloy, in the present work, only gehlenite (Ca_2_Al_2_SiO_7_) and magnesium spinel (MgAl_2_O_4_) were found as corrosion reaction products. It is worth noting that thermodynamic calculations were performed, and this formation aligns with the thermodynamic predictions previously mentioned in Table 3.

The interfacial reaction between the molten Al–Mg alloy and the anorthite coating proceeds via a solid-state cation interdiffusion and interfacial redox mechanism rather than a direct elemental dissolution process. Anorthite possesses a triclinic silicate framework in which Si^4+^ (ionic radius of 0.040 nm) and Al^3+^ form ordered tetrahedra [29]. Driven by chemical potential gradients at 1000 °C, interdiffusion occurs between the molten alloy (Al and Mg) and the coating (Ca and Si). Specifically, Si^4+^ cations in the SiO_4_ tetrahedra are substituted by Al^3+^ from the molten metal, transforming SiO_4_ into AlO_4_ tetrahedra and releasing Si into the liquid alloy. Concurrently, Ca^2+^ diffuses outwards, while Mg^2+^ penetrates the surface layer, leading to the local crystallization of thermodynamically stable gehlenite (Ca_2_Al_2_SiO_7_) and magnesium spinel (MgAl_2_O_4_), as confirmed by TEM and XPS analyses.

This was observed using the transmission electron microscopy (TEM) analysis, detecting the formation of gehlenite at the interface when both Si and Al–Ca–Si precipitates were present in the Al alloy channels (Figure 11).

The presence of these precipitates in the immediate vicinity of the anorthite indicates that once the saturation solubilities of Ca and Si have been reached, further diffusion into the bulk Al alloy may be suppressed, reducing or even stopping the Si^4+^–Al^3+^ interdiffusion rate. This suggests that the primary factor controlling phase evolution is the kinetics of the transfer of dissolved Si from the Al alloy channels toward the bulk of the alloy.

Table 5 shows the comparison between the theoretical and experimental interplanar distance values for the anorthite compound. It can be observed that the measured values are in close agreement with each other, which suggests that the analyzed zone corresponds to a single-phase of the anorthite compound.

Table 6 shows the comparison between the theoretical and experimental interplanar distance values for the gehlenite compound. It can be observed that the measured values are in close agreement with each other, which suggests that the analyzed zone corresponds to the gehlenite compound.

**Figure 12:**
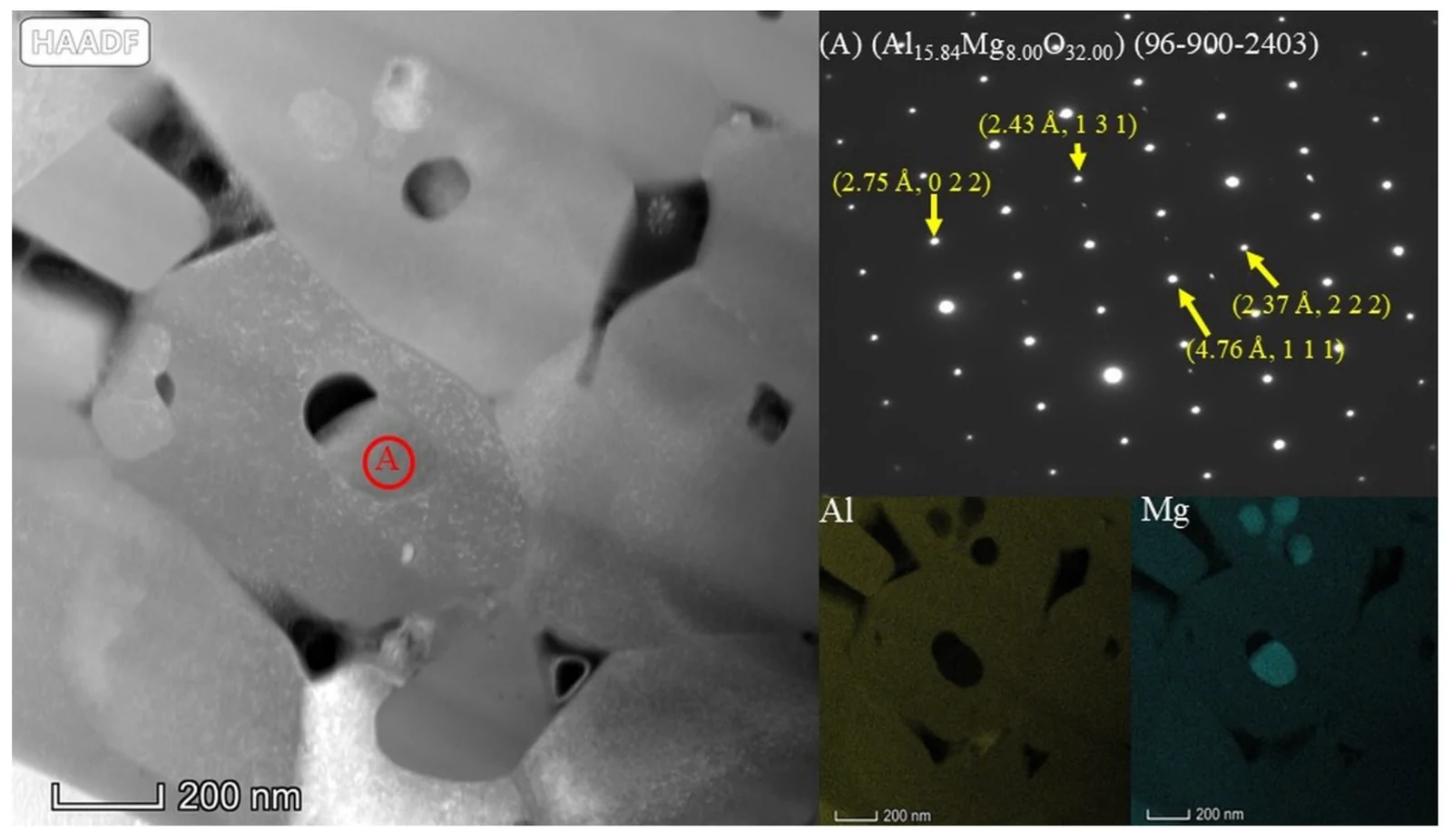
Transmission electron microscopy (TEM) analysis of the anorthite coating surface.

Figure 12 shows the surface of the anorthite coating, which was in contact with molten aluminum. In area (A), the transmission electron microscopy (TEM) analysis suggests the presence of magnesium spinel (MgAl_2_O_4_), which is in agreement with the thermodynamic predictions. This indicates that magnesium diffusion effectively occurred on the surface of the ceramic coating, leading to the formation of beneficial reaction products. The presence of compounds such as gehlenite and spinel favors the chemical behavior of the ceramic surface, providing the refractory with corrosion resistance properties, as these are stable compounds that are not reduced in the presence of liquid aluminum.

In accordance with the mechanistic framework developed by Adabifiroozjaei et al. [29,30], the interfacial reaction between the molten Al–Mg alloy and the anorthite coating occurs via a sequential two-stage interdiffusion process:

Initial stage (Ca^2+^–Mg^2+^ interdiffusion): Upon initial contact at high temperature (1000°C), rapid interdiffusion occurs between Ca^2+^ ions from the silicate lattice and active Mg^2+^ ions from the molten alloy (involving intermediate calcium aluminates such as CA_2_). This drives the early nucleation and growth of the interfacial magnesium spinel (MgAl_2_O_4_) protective layer.

Secondary stage (Ca^2+^–Al^2+^ interdiffusion): As holding time progresses, enhanced evaporation of volatile Mg from the molten metal gradually lowers the local Mg activity at the liquid/solid interface. This reduction in Mg activity shifts the diffusion equilibrium, favoring a transition toward Ca^2+^–Al^2+^ interdiffusion and Si^4+^↔Al^3+^ tetrahedron substitution, which promotes the crystallization of gehlenite (Ca_2_Al_2_SiO_7_) and MgAl_2_O_4_.

This sequential mechanism fully accounts for the coexistence of both gehlenite and magnesium spinel detected at the corroded interface by TEM (Table 6 and Table 7) and XPS core-level analysis (Table 8 and Table 9), confirming the establishment of a stable, non-reactive protective barrier against liquid metal penetration.

In the present work, thermodynamic calculations (Table 3) indicate that the formation of magnesium spinel (MgAl_2_O_4_) is thermodynamically favorable (ΔG < 0) during the reaction between the anorthite coating and the molten Al–Mg alloy. From a kinetic perspective, the elevated testing temperature (1000 °C) and the high local activity of Mg promote rapid interdiffusion of Mg^2+^ and Al^3+^ species across the interfacial boundary layer. This kinetic pathway, combined with thermodynamic driving forces, leads to the continuous crystallization and stabilization of the MgAl_2_O_4_ phase at the interface, as definitively confirmed by transmission electron microscopy (TEM) (selected area electron diffraction (SAED) interplanar distances, Table 7) and X-ray photoelectron spectroscopy (XPS) (binding energy core levels, Table 8 and Table 9).

Table 7 shows the comparison between the theoretical and experimental interplanar distance values for the magnesium spinel compound. It can be observed that the measured values are in close agreement with each other, which suggests that the analyzed zone corresponds to magnesium spinel compound.

### 3.6. X-Ray Photoelectron Spectroscopy (XPS) Analysis of the Anorthite Coating

**Figure 13:**
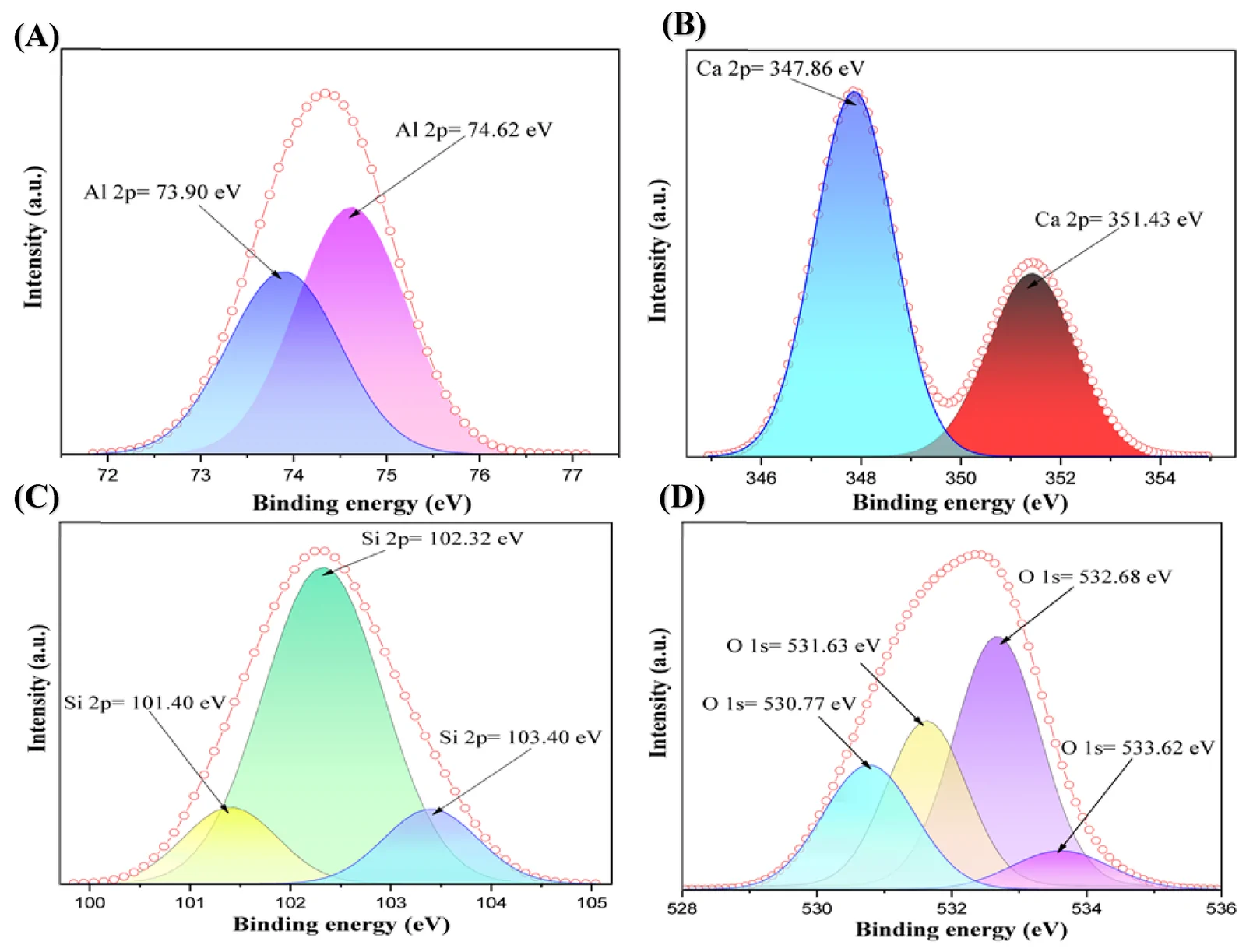
X-ray photoelectron spectroscopy (XPS) spectrum of anorthite coating: (**A**) Al 2p spectrum, (**B**) Ca 2p spectrum, (**C**) Si 2p spectrum, (**D**) O 1s spectrum.

Figure 13 shows the anorthite (CaAl_2_Si_2_O_8_) X-ray photoelectron spectroscopy (XPS) spectrum, in which energy calibration was performed using the C 1s = 284.8 eV peak as a reference, a standard value in X-ray photoelectron spectroscopy (XPS). The peaks correspond exclusively to Ca 2p, Al 2p, Si 2p, and O 1s, which indicates the formation of only the anorthite phase.

Pattern A shows an Al 2p peak (74.62 eV), indicating that aluminum is in its +3 oxidation state (Al^3+^), tetrahedrally coordinated within the lattice (AlO_4_). Its position is consistent with aluminosilicates such as anorthite and feldspars [31]. A secondary peak at 73.90 eV is observed, attributed to surface sites of octahedral aluminum or the presence of Al_2_O_3_/Al–OH formed during the corrosion test.

Pattern B displays a doublet in the Ca 2p region, with peaks centered at Ca 2p_3/2_ = 347 eV and Ca 2p_1/2_ = 351.0 eV, corresponding to the characteristic spin-orbit splitting of calcium in the Ca^2+^ state in octahedral coordination within the calcium aluminosilicate lattice (anorthite) [32]. The Ca 2p spin-orbit doublet represents not a new bond or contamination, but rather a natural splitting of the same electronic level of calcium.

Pattern C shows the Si 2p peaks (~102.3–103.0 eV), which correspond to silicon in the +4 oxidation state (Si^4+^) forming SiO_4_ tetrahedra. The value around 102.3 eV confirms the presence of fully oxidized silicate; the shift towards 103 eV may be due to a slightly more electronegative environment (Si–O–Al) [33].

Pattern D shows the O 1s peaks (~531.0–531.8 eV) which are typical of bridging oxygens O–Si–Al and O–Ca in the feldspathic network [32]. Secondary peaks near 532.5 and 533 eV are associated with the oxygen present in the surface in the form of hydroxyl groups (–OH) or adsorbed carbonates (C=O) [32].

**Figure 14:**
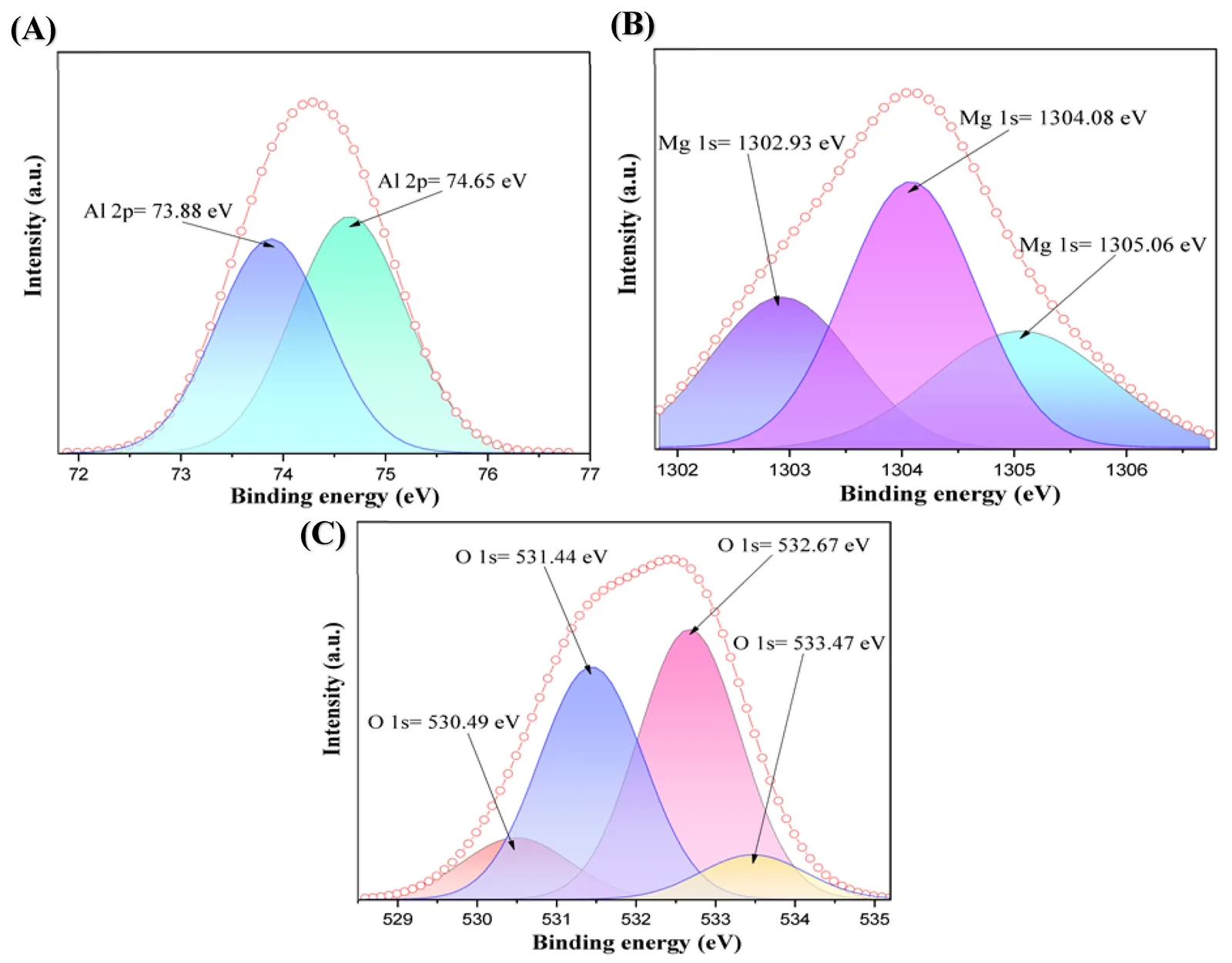
High-resolution core-level XPS spectra of the interface between the anorthite coating and the Al-5Mg alloy after corrosion testing: (**A**) Al 2p spectrum showing Al_2_O_3_ and MgAl_2_O_4_ species, (**B**) Mg 1s spectrum showing Mg(OH)_2_, MgAl_2_O_4,_ and MgO chemical states, and (**C**) O 1s spectrum detailing the oxygen bonding environments for MgO, MgAl_2_O_4,_ Mg(OH)_2_, and Al_2_O_3_.

Figure 14 shows the X-ray photoelectron spectroscopy (XPS) results of the interface between the anorthite coating and the alloy after corrosion testing. In this region, the obtained peaks correspond to reaction products formed by the reduction of oxides caused by magnesium. The binding energies were adjusted using the C 1s = 284.8 eV peak as a reference. The Mg 1s and O 1s energies for the values corresponding to magnesium hydroxide (Mg(OH)2) are 1302.93 eV and 532.67 eV, respectively. According to the literature, the peaks corresponding to magnesium hydroxide and O 1s are reported at 1302 eV and 532.5 eV, respectively [34,35]. Furthermore, the peaks of Mg 1s and O 1s, which indicate the formation of magnesium oxide (MgO), confirm that a reduction reaction occurred. These values are found at 1305.06 eV and 530.49 eV, while, in the literature, magnesium oxide is reported at values of Mg 1s = 1305.8 eV and O 1s = 530.4 eV [36,37].

The Mg 1s, Al 2p, and O 1s binding energies confirm the formation of magnesium spinel (MgAl_2_O_4_), with recorded values at 1304.08, 74.64, and 531.44 eV, respectively. In the literature, magnesium spinel is reported at values of 1304.5, 75, and 531.5 eV [36,38,39]. Furthermore, the Al 2p and O 1s energies are consistent with alumina (Al_2_O_3_) formed by the reduction process triggered during the corrosion test. These values were found at 73.88 and 533.47 eV, whereas the literature reports values of 73.8 eV and 533 eV for Al 2p and O 1s, respectively [40,41].

Table 8 presents a systematic comparison between the experimental binding energy values obtained in this work (Test) for the oxygen O 1s core level and the corresponding values reported in the scientific literature (Litt). The analysis of the O 1s signal on the surface of the corroded coating reveals the coexistence of four distinct oxidized phases at the interface:

1. Magnesium oxide (MgO): identified by a peak at 530.49 eV (Test), which is in close agreement with the literature value of 530.4 eV [37]. This compound is generated as a direct product of the initial reduction process of the oxides in the ceramic coating driven by the active magnesium from the alloy.
2. Magnesium spinel (MgAl_2_O_4_)/alumina (Al_2_O_3_): The peak at 531.44 eV is assigned to lattice oxygen within the aluminate/spinel framework and the alumina matrix (531.1 eV) [39,41]. This equilibrium phase (MgAl_2_O_4_) is crucial for the material performance, as it provides stability against molten aluminum attack. Alumina (Al_2_O_3_) confirms the oxidation of metallic aluminum during interfacial reduction reactions.
3. Magnesium hydroxide (Mg (OH)_2_): recorded at 532.67 eV (Test) compared to 532.5 eV [35] in the literature, which is attributed to surface hydration or partial hydrolysis of the magnesium species exposed to the environment prior to measurement.
4. Surface carbonates (MgCO_3_)/adsorbed species: located at 533.47 eV, corresponding to surface carbonate species and adsorbed oxygen/water resulting from atmospheric exposure of MgO (533.0 eV) [37].

**Table 8:** Binding energies for oxygen O 1s of the anorthite coating on the surface area after the corrosion test (magnesium spinel MgAl_2_O_4_ formation).

Compound/Assignment	O 1s (eV) Experimental	O 1s (eV) Literature	Refs.
MgO (Lattice Oxygen)	530.49	530.4	[37]
MgAl_2_O_4/_Al_2_O_3_ (Lattice Oxygen)	531.44	531.1	[39,41]
Mg (OH)_2/_Surface Hydroxyls {-OH}	532.67	532.5	[35]
{MgCO_3_)/Adsorbed Carbonates and Oxygen	533.47	533.0	[37].

Table 9 summarizes the high-resolution spectra corresponding to the magnesium (Mg 1s) and aluminum (Al 2p) metallic cations at the interface after the corrosion test, contrasting the experimental results (Test) with theoretical references (Litt). These data confirm the oxidation states and atomic coordination of the new protective phases synthesized in situ on the refractory surface.

For Mg 1s: Three well-defined chemical states were identified. The peak at 1305.06 eV corresponds to MgO (Litt: 1305.8 eV, [36]), the peak at 1304.08 eV is assigned to the tetrahedral structure of magnesium spinel (MgAl_2_O_4_) (Litt: 1304.5 eV, [36]) and the peak at 1302.93 eV belongs to (Mg(OH)_2_) (Litt: 1302 eV, [34]).

For Al 2p: The aluminum peaks corroborate the coordination environments of the Al^3+^ cation. The value of 73.88 eV confirms the presence of alumina (Al_2_O_3_) (Litt: 73.8 eV, [40]) as a product of thermic reduction. On the other hand, the peak shifted to 74.65 eV is associated with octahedrally coordinated aluminum within the crystal lattice of the spinel (MgAl_2_O_4_) (Litt: 75.0 eV, [38]).

Overall, the excellent correspondence of the cation-binding energies (Mg 1s and Al 2p) with those of oxygen (O 1s) in both tables provides experimental evidence that the spontaneous reduction reactions lead to the protective passivation of the coating through the crystallization of magnesium spinel and alumina.

**Table 9:** Binding energies for magnesium and aluminum (Mg 1s, Al 2p) of the anorthite coating on the surface area after the corrosion test (magnesium spinel MgAl_2_O_4_ formation).

Compound	Mg1s eV (Mg (OH)_2_)		Mg1s eV (MgAl_2_O_4_)		Mg1s eV (MgO)		Al2p eV	
	Test	Litt.	Test	Litt.	Test	Litt.	Test	Litt.
MgO/Al_2_O_3_	-	-	-	-	1305.06	1305.8 [36]	73.88	73.8 [40]
MgAl_2_O_4_	-	-	1304.08	1304.5 [36]	-	-	74.65	75 [38]
Mg (OH)_2_	1302.93	1302 [34]	-	-	-	-		

### 3.7. Study of Static Sessile Drop Wettability Tests

**Figure 15:**
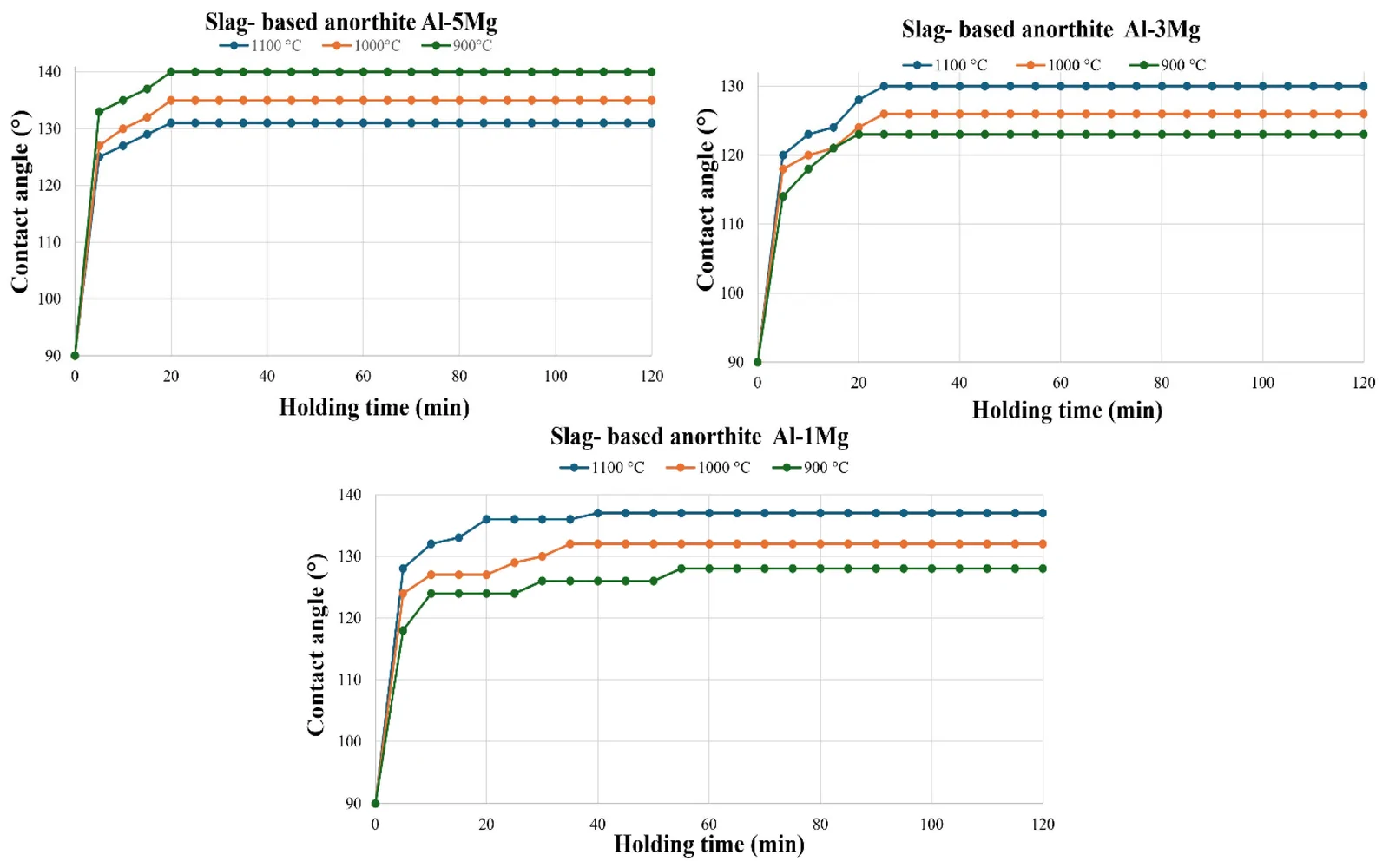
Contact angle vs. time for wettability tests with Al-5Mg, Al-3Mg, and Al-1Mg alloys on slag-based anorthite coating at 900, 1000, and 1100 °C.

In this section, the results following the wettability test of the samples in contact with the Al-1Mg, Al-3Mg, and Al-5Mg alloys at 900 °C, 1000 °C, and 1100 °C will be discussed. Figure 15 presents the contact angle plots for the anorthite (CaAl_2_Si_2_O_8_) samples. It is observed that, at higher temperatures and lower Mg concentrations (Al-1Mg), the contact angle increases (reaching 138° at 1100 °C). At higher Mg concentrations (Al-5Mg) and higher temperatures, the contact angle is lower (131° at 1100 °C). Since all reported angles are greater than 90°, the coating is classified as non-wetting. It should be noted that in all cases, an equilibrium state is reached after a few minutes.

When comparing the performance of the developed coating with conventional refractory materials, a substantial improvement in the wetting resistance against molten aluminum was demonstrated. The results obtained in this work for the dross-based synthesized anorthite coating reveal higher contact angles than those formed on mullite, reaching values close to 140° (as observed in Figure 15, with a maximum angle of 138° for the Al-1Mg alloy at 1100 °C and 140° for the Al-5Mg alloy at 900 °C). In contrast, Ibarra and co-workers [42] reported that a commercial mullite compound exhibits significantly lower contact angles, within the range of 120°, under similar interaction conditions with aluminum alloys. This increase in the contact angle (from 120° to values close to 140°) is of great technological significance. It indicates that the anorthite coating possesses lower surface energy and a better non-wetting behavior. This protective performance is directly linked to the reactive nature of the system: the in situ formation of thermodynamically stable phases at the interface, such as magnesium spinel and gehlenite, progressively increases the droplet contact angle until stabilization is reached. As free silica in commercial mullite tends to be reduced by the molten aluminum alloy (degrading the refractory and promoting wetting), the anorthite coating acts as a highly effective chemical and physical barrier, preventing infiltration of the aluminum and successfully protecting the structural integrity of the mullite substrate.

## 4. Conclusions

The formation of anorthite (CaAl_2_Si_2_O_8_) compounds for coatings was successfully achieved using purified aluminum dross.

Corrosion tests confirmed that the coatings exhibit thermal reactions at the interface, as reduced metallic silicon originating from the anorthite coating was identified within the Al-Mg alloy. However, these reactions do not degrade or detach the coating; instead, they result in the formation of reaction products such as gehlenite and magnesium spinel, consistent with thermodynamic predictions. Consequently, the coating is effective in protecting the mullite substrate from thermal reduction reactions.

Transmission electron microscopy (TEM) analysis, through the comparison of measured interatomic distances with corresponding reference patterns, confirms the formation of gehlenite and magnesium spinel on the surface of the anorthite coating following the corrosion tests. This transformation is highly beneficial, as the resulting spinel is chemically stable and resistant to further degradation by Al-Mg alloys.

The X-ray photoelectron spectroscopy (XPS) test results were compared with theoretical data, finding correlations between the binding energy values of the elements, which confirms the formation of slag-based anorthite and the presence of magnesium spinel.

Rietveld structural refinement proves that a single-phase anorthite compound was obtained from purified aluminum dross, as all diffraction peaks are representative of the anorthite reference pattern, and no other phase is present.

In the wettability test, it was found that all contact angles began at 90°, which is defined as the intrinsic wettability angle of the material. The contact angle varied over time, increasing until reaching a state of stability. This increase is primarily due to the fact that the study systems are reactive systems where the contact surface undergoes changes in its chemical composition. The reaction products generated at the interface directly influence the final contact angle.

The results of the thermodynamic analysis show that the compound studied for coatings is not stable in contact with an Al-Mg alloy at high temperatures. However, the corrosion results demonstrate that the coatings did not degrade completely and successfully protected the substrate; this behavior is closely related to the observed wetting angles.

## Figures and Tables

**Table 1:** Chemical analysis results (as oxides) of unpurified and purified dross obtained by AA and/or ICP.

Composition (wt.%)									
Compound	Al_2_O_3_	Al	CaO	Fe_2_O_3_	K_2_O	MgO	Na_2_O	ZnO	SiO_2_
Al dross	60.72	13.14	0.30	0.46	0.79	3.93	5.27	0.07	15.19
Purified dross	79.47	3.63	0.01	0.24	0.32	4.53	0.88	0.01	10.37

**Table 2:** Amount of the precursors used to synthesize anorthite (stoichiometric amount, wt.%).

Amount (wt.%)				
Compound	Dross	Al_2_O_3_	SiO_2_	CaCO_3_
CaAl_2_Si_2_O_8_	26.19	9.32	34.42	30.07

**Table 3:** Thermodynamic systems of coatings in contact with molten Al and Al-Mg alloy.

Reaction of Commercial Mullite with Molten Aluminum	
System 1	Equilibrium reaction products.
Al_6_Si_2_O_13_, SiO_2_, Al 1000 °C	Si, Al_2_O_3_
Reaction of commercial mullite with Al-Mg alloy	
System 2	Equilibrium reaction products.
Al_6_Si_2_O_13_, SiO_2_, Al, Mg 1000 °C	MgAl_2_O_4_, Si
Reaction of commercial mullite with anorthite	
System 3	Equilibrium reaction products.
Al_6_Si_2_O_13_, SiO_2_, CaAl_2_Si_2_O_8_ 1400 °C	Al_6_Si_2_O_13_, SiO_2_, CaAl_2_Si_2_O_8_
Reaction of anorthite with molten aluminum	
System 4	Equilibrium reaction products.
CaAl_2_Si_2_O_8_, Al 1000 °C	Si, Al_2_O_3_, Ca_2_Al_2_SiO_7_
Reaction of anorthite with molten Al-Mg alloy	
System 5	Equilibrium reaction products.
CaAl_2_Si_2_O_8_, Al, Mg 1000 °C	Si, MgAl_2_O_4_, Ca_2_Al_2_SiO_7_

**Table 4:** Chemical analysis of the actual composition of the Al-Mg alloys (wt.%).

Alloy	Element (Mg)	Element (Al)
Al-1Mg	1.23	98.77
Al-3Mg	3.10	96.90
Al-5Mg	5.36	94.64

**Table 5:** Interplanar distance values of anorthite.

Measured Values of the Interplanar Spacings			
Compound (CaAl_2_Si_2_O_8_)	Plane	Theoretical distance (Å)	Experimental distance (Å)
Anorthite (96-210-7236)	1 1 1	6.52	6.60
	2 2 2	3.26	3.29
	−2 0 2	3.19	3.24
	3 1 3	2.59	2.43

**Table 6:** Interplanar distance values of gehlenite.

Measured Values of the Interplanar Spacings			
Compound (Ca_2_Al_2_SiO_7_)	Plane	Theoretical distance (Å)	Experimental distance (Å)
Gehlenite (96-101-1003)	1 1 1	3.71	3.57
	2 1 1	2.85	2.77
	2 1 2	2.04	2.00
	2 2 2	1.85	1.85
	3 2 3	1.32	1.33

**Table 7:** Interplanar distance values for magnesium spinel.

Measured Values or the Interplanar Spacings			
Compound (Al_15_._84_Mg_8_O_32_)	Plane	Theoretical distance (Å)	Experimental distance (Å)
Spinel (96-900-2403)	1 1 1	4.66	4.76
	0 2 2	2.85	2.75
	1 3 1	2.43	2.43
	2 2 2	2.33	2.37

## Data Availability

The original contributions presented in this study are included in the article. Further inquiries can be directed to the corresponding authors.
